# Clinical Fellowships in Surgical Training: Analysis of a National Pan-specialty Workforce Survey

**DOI:** 10.1007/s00268-013-1949-1

**Published:** 2013-02-20

**Authors:** J. E. F. Fitzgerald, J. A. Milburn, G. Khera, R. S. M. Davies, S. T. Hornby, C. E. B. Giddings

**Affiliations:** Association of Surgeons in Training, 35-43 Lincoln’s Inn Fields, London, WC2A 3PE UK

## Abstract

**Background:**

Fellowship posts are increasingly common and offer targeted opportunities for training and personal development. Despite international demand, there is little objective information quantifying this effect or the motivations behind undertaking such a post. The present study investigated surgical trainees’ fellowship aims and intentions.

**Methods:**

An electronic, 38-item, self-administered questionnaire survey was distributed in the United Kingdom via national and regional surgical mailing lists and websites via the Association of Surgeons in Training, Royal Surgical Colleges, and Specialty Associations.

**Results:**

In all, 1,581 fully completed surveys were received, and 1,365 were included in the analysis. These represented trainees in core or higher training programs or research from all specialties and training regions: 66 % were male; the mean age was 32 years; 77.6 % intended to or had already completed a fellowship. Plastic surgery (95.2 %) and cardiothoracic (88.6 %) trainees were most likely to undertake a fellowship, with pediatrics (51.2 %), and urology (54.3 %) the least likely. Fellowship uptake increased with seniority (*p* < 0.01) and was positively correlated (*p* = 0.016, *r* = 0.767) with increasing belief that fellowships are necessary to the attainment of clinical competence, agreed by 73.1 %. Fellowship aims were ranked in descending order of importance as attaining competence, increasing confidence, and attaining subspecialist skills.

**Conclusions:**

Over three-quarters of trainees have or will undertake a clinical fellowship, varying with gender, specialty, and seniority. Competence, confidence, and subspecialty skills development are the main aims. The findings will influence workforce planning, and perceptions that current training does not deliver sufficient levels of competence and confidence merit further investigation.

## Introduction

Clinical fellowships for surgical trainees are common worldwide and can offer targeted opportunities for additional training and personal development. Typically lasting 6 months to 1 year, these optional posts are frequently undertaken toward the end of training at centers offering supervised subspecialty clinical experience.

The role of the fellow may differ widely between individual institutions, and the overall role of fellowship training differs between countries. In the United States, where fellowship posts are well established, annual reviews are required by the Accreditation Council for Graduate Medical Education (ACGME) in addition to their yearly resident-fellow national survey.

In the United Kingdom perceptions remain that while many high-quality fellowship posts exist, some merely maintain rota staffing levels outside of nationally agreed terms and conditions of service for junior doctors. In addition there are concerns that fellows may cherry-pick operative cases to the detriment of local trainees’ experience. An optional fellowship accreditation system is in early development in the UK following concerns regarding the absence of quality assurance for these posts [1]. With the exception of a few nationally supported posts in defined programs (e.g., laparoscopic colorectal surgery) [2] and centrally funded “Interface Fellowships” [3] there is currently no register of opportunities and no centralized application system (Interface Fellowships offer themed training in specific areas of practice where different surgical specialities “interface”—e.g., cleft palate fellowships for plastic, pediatric, maxillofacial, or otorhinolaryngology trainees).

To-date no pan-specialty national study has sought to investigate the demand or motivations to pursue surgical fellowship training. The aim of the present study was to establish the motivating factors behind undertaking such a post, report speciality and demographic variations, and provide objective information regarding demand in each surgical specialty, together with views on the centralization of fellowship applications.

## Methods

### Defining clinical fellowship training

For the purposes of this study a “fellowship” in surgery was defined as “an optional, additional period of clinical work undertaken within a defined specialty or subspecialty area by a surgeon not yet appointed to a substantive consultant position, and for whom this additional period is not a mandatory requirement of their training program.”

### Participants and setting

**Fig. 1:**
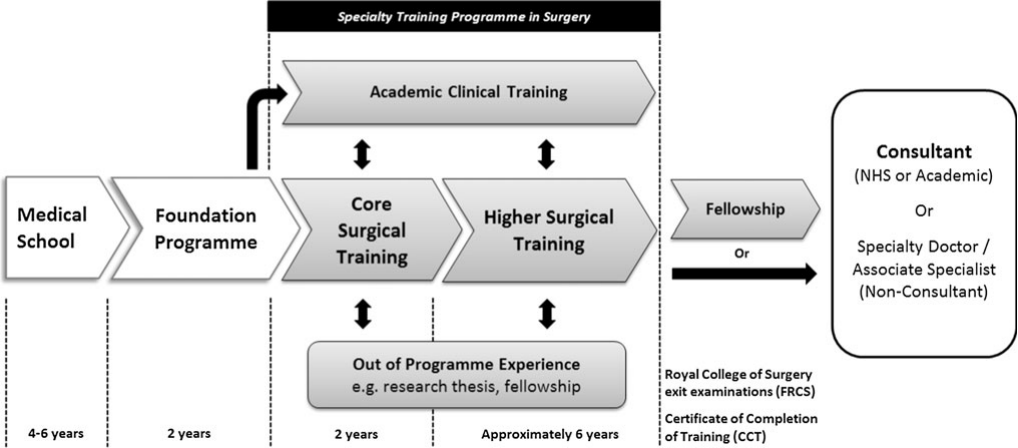
Schematic overview of surgical training in the United Kingdom. Adapted from [4]

In the UK, following completion of an undergraduate medical degree all graduates enter a two-year generic postgraduate training program (the “Foundation Programme”). Following this, doctors wishing to pursue a career in surgical specialities apply through a UK-wide national competitive selection process into a “Core Training” program lasting two years. Core Training may be generic or themed around a particular surgical speciality, and is followed by competitive application for a “Speciality Training” (ST) program. The ST schemes last up to six years and provide dedicated training in one of the nine defined surgical specialities (general, orthopedics and trauma, urology, pediatrics, otorhinolaryngology, plastic, maxillofacial, cardiothoracic, and neurosurgery). During this period trainees will rotate between hospitals and supervising consultants, usually at 6-monthly intervals. At the end of Speciality Training a doctor receives a Certificate of Completion of Training (CCT) upon successful demonstration of the required competencies, including passing an exit examination set by the Royal Surgical Colleges. Clinical fellowship posts are typically undertaken toward the end of training by applying for “out of program experience” prior to completion of the training program (pre-CCT), or after formal training has been completed (post-CCT) prior to taking up a consultant post. A schematic overview of this training is provided in Fig. 1. More detail regarding the relevant structure and pathways through surgical training in the UK has previously been described [4]. Across the UK, there are currently 946 Core Trainees and 4,393 Specialty Trainees registered in surgical training programs (based on 2012 figures from the Joint Committee on Surgical Training, [http://www.jchst.org/]).

### Questionnaire

An electronic, 38-item, self-administered questionnaire survey was developed exploring demographics, career aims, and factors influencing motivations for pursuing a clinical fellowship. This consisted of free text, binomial and 5-point Likert scale responses. The questionnaire was piloted by over 30 surgical trainees of varying grades of seniority and specialty to ensure content and face validity. The feedback received was used to further refine the question items both in terms of content and wording in order to remove ambiguity and ensure question neutrality. Test–retest and inter-observer reliability were not appropriate to establish with this study design. Given the range of different constructs measured in the questionnaire (e.g., training, demand, organization, and aims) internal consistency calculations were not performed.

Junior doctors in surgical training (i.e., pre-CCT) in the UK were invited to participate in this non-mandatory survey through surgical mailing lists and websites by the Association of Surgeons in Training (ASiT), Royal Surgical Colleges, and Specialty Associations. Responses were collected through the SurveyMonkey web-survey portal (SurveyMonkey.com, LLC, Palo Alto, CA). To ensure data integrity, Internet protocol (IP) address blocking was used to prevent multiple submissions. Answer randomization was enabled where appropriate in order to minimize order bias. The online questionnaire survey was open from June through November 2011 and was re-publicised at regular intervals in order to maximize the response rate.

This study was undertaken by the Association of Surgeons in Training as part of a national workforce survey for doctors in surgical training. The authors gave due consideration to the ethical dimensions of this anonymous non-mandatory questionnaire survey, and no concerns were identified. Completion of the questionnaire was taken as consent to participate.

### Data analysis

Analysis of results was undertaken with the Statistical Package for the Social Sciences, SPSS 17.0 (SPSS Inc. Chicago, IL). Categorical data were analyzed for significance with the chi-squared test, and the nonparametric Spearman correlation coefficient was used to investigate for significant correlations.

## Results

Of 1,710 questionnaires submitted, 1,581 were appropriately completed sufficient for further analysis. From these, medical student (*n* = 14), Foundation Programme (*n* = 74), post-CCT (*n* = 51), and non-training grade (*n* = 77) responses were excluded unless otherwise stated, leaving 1,365 responses to be included in the analysis. These exclusions focused the review solely on trainees who were part of recognized training programs or who were taking time out from such a program for research.

### Demographics

Overall, 906 of the 1,365 respondents were men (66.4 %) and the mean age was 32 years (range: 25–54 years). The demographics of respondents are summarized in Table 1. Responses were received from all 19 regional postgraduate medical training organizations (NHS deaneries) covering England, Northern Ireland, Scotland, and Wales, plus the Defence Deanery (Table 2). Responses were received from physicians representing all 9 surgical specialties (Table 3).

**Table 1:** Demographics of survey respondents and fellowship intentions

Grade of training	Number of respondents	Mean age (years)	Gender (male %)	Time in surgical training to-date (years)	Have you already, or do you intend to, complete a clinical fellowship? Yes (%)	Do you feel fellowships are necessary to allow attainment of a level of clinical competence necessary for independent practice in your specialty? Yes (%)
CT Year 1	132	28.2	62.9	1.2	67.4	68.9
CT Year 2	175	29.2	64.6	2.2	73.1	67.4
StR 3–4/SpR 1–2	328	31.9	69.8	5.3	76.7	79.9
StR 5–6/SpR 3–4	339	34.1	66.1	8.1	81.7	72.6
StR 7–8/SpR 5–6	258	36.2	64.7	10.4	81.7	68.6
Research Fellow	133	31.3	67.7	5.1	85.0	81.2
Mean	–	31.8	66.4	–	77.6	73.1

*CT* Core Trainee (formerly Senior House Officer [SHO]); *StR/SpR* Specialist Registrar/Specialty Registrar

In the UK, Specialty Registrar (StR) grade numbering continues on from Core Training and is replacing the old Specialist Registrar (SpR) grade

**Table 2:** Fellowship intentions and perceptions by surgical specialty

Specialty	Number of respondents	Have you already, or do you intend to, complete a clinical fellowship? Yes (%)	Do you feel fellowships are necessary to allow attainment of a level of clinical competence necessary for independent practice in your specialty? Yes (%)
Cardiothoracic	35	31 (88.6)	25 (71.4)
ENT	114	96 (84.2)	75 (65.8)
General surgery	667	512 (76.8)	492 (73.8)
Neurosurgery	86	75 (87.2)	58 (67.4)
OMFS	12	7 (58.3)	5 (41.7)
Paediatric surgery	43	22 (51.2)	20 (46.5)
Plastic surgery	84	80 (95.2)	75 (89.3)
Trauma and orthopedics	237	202 (85.2)	198 (83.5)
Urology	81	44 (54.3)	53 (65.4)

**Table 3:** Fellowship intentions and perceptions by gender

Response	Have you already, or do you intend to, complete a clinical fellowship?		Do you feel fellowships are necessary to allow attainment of a level of clinical competence necessary for independent practice in your specialty?	
	Male [*N* (%)]	Female [*N* (%)]	Male [*N* (%)]	Female [*N* (%)]
Yes	737 (81.4)	335 (73)	667 (73.6)	335 (73.0)
No	46 (5.1)	19 (4.1)	144 (15.9)	48 (10.5)
Unsure	122 (13.5)	105 (22.9)	95 (10.5)	76 (16.6)

### Demand for clinical fellowship training in surgery

Across all grade of trainee, an average of 77.6 % intended to, or had already completed a clinical fellowship. Demand increased in line with seniority from 67.4 % of first-year Core Trainees to 81.7 % of year 5–6 registrars (Table 1). Fellowship intentions also varied considerably between surgical specialties (Table 2), with plastic surgery trainees most frequently stating an intention to undertake a fellowship (95.2 %) and pediatric surgery trainees least frequently (52.1 %). Men were more likely than women to plan on undertaking a fellowship (Table 3).

### Clinical fellowships and clinical competence

**Fig. 2:**
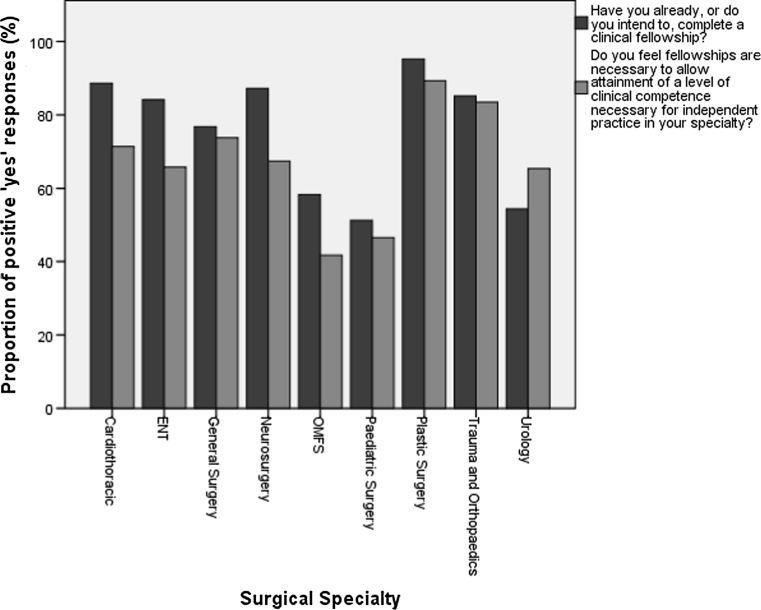
Fellowship intentions and perceptions by surgical specialty

In addition to establishing demand for fellowship positions, trainees were asked whether they perceived that fellowship training was necessary to allow attainment of clinical competence necessary for independent practice in their specialty. Overall, 73.1 % indicated that fellowship training was necessary, lower than the 77.6 % who intended to, or who had already completed a fellowship. Analyzing these results by specialty choice, there was a positive significant correlation (*p* = 0.016, *r* = 0.767) between intention to undertake a fellowship and the belief that fellowship training was necessary to attain clinical competence. Urology was the only specialty where the proportion that believed fellowship training was necessary to attain clinical competence was higher than the number of trainees intending to, or who had already completed a fellowship. These results are summarized by specialty in Table 2 and Fig. 2. The proportions of male and female trainees who believed fellowship training was necessary to attain clinical competence were similar (74 % of male trainees versus 73 % of females). These responses are summarized in Table 3.

### Selection in clinical fellowship training

Given the absence of uniform accreditation or quality assurance for fellowship posts in the UK, coupled with the lack of a central register of opportunities, trainees were asked whether the ad-hoc system currently in place should be formalized. Despite the high proportion of trainees who believed fellowship training was necessary to attain clinical competence, only 19.9 % believed that a fellowship post should be compulsory prior to appointment to a consultant post, ranging from 11.4 % in otolaryngology (ENT) and cardiothoracic surgery to 31.6 % in trauma and orthopedic surgery. Higher proportions of male trainees believed fellowship posts should be compulsory, with the exception of plastic, cardiothoracic and maxillofacial surgery, which were equal in opinion between male and female trainees.

Formalization of fellowship applications through a central national selection system was not supported by the majority of respondents, with only 25.2 % agreeing that they would wish to see the introduction of such a system. Were formalized applications to be introduced, the majority of trainees wished administration of the application process to remain within the surgical profession, with 36.6 % indicating this would be an appropriate role for the Surgical Royal Colleges, 28.0 % preferring oversight by speciality organizations, 19.5 % preferring regional postgraduate deaneries, and only 10.2 % preferring employing organizations.

### Aims of undertaking clinical fellowship training

The majority of trainees cited increasing confidence (82.9 %), developing competence (81.5 %), and attaining super-specialist skills (79.3 %) as the primary reasons for wishing to undertake a fellowship post (Table 4). Free text responses of other aims were also collected, and 30 comments were received. The majority of these noted the need for fellowships to be more than attaining competence, which many cited as being the remit of pre-CCT specialty training programs. Many also argued against the formalization of fellowship training with centralized applications, citing the high-profile failure of the UK Medical Training Application Service (MTAS) program in 2007 (aborted following national concerns surrounding an inadequately secured, overloaded, and poorly administered online application system with flawed marking criteria). A representative sample of these comments is provided in Table 5.

**Table 4:** Fellowship aims ranked in order of cited importance

Fellowship aim	Number responding with aim rated as “important” [*N* (%)]
Increase confidence	733 (82.9)
Competence	923 (81.5)
Attain super-specialist skills	808 (79.3)
CV and portfolio building	343 (40.6)
Making contacts	291 (37.2)
Non-clinical purposes (e.g., research)	193 (24.0)

**Table 5:** Representative free text comments regarding fellowship aims

“Highly individual. the point would be for individual plans”
“Character, mind and personal development”
“Period of no on call”
“Competence should have already been attained by the end of ST, otherwise what’s the point of ST [higher specialty] training? Fellowships should be something ‘extra’ and ‘beyond’ ST training”
“Seeing how other people practice in other units outside your own”
“They should be unnecessary”
“They should not be formalized”
“Attainment of competence to practice as a generalist post CCT should be the role of specialty training—if it cannot meet that, it needs to be lengthened”

## Discussion

This study reports the first national pan-specialty survey of trainees in relation to clinical fellowship posts in surgical training. Overall, more than three quarters of respondents planned to undertake, or had already completed a fellowship post, highlighting the current popularity of these positions. Despite differences in training programs and the role played by fellowships in developing specialty practice, this proportion is similar to that reported in recent North American studies [5–7].

The demand for clinical fellowship experience increased in line with trainee seniority. The reasons for this are likely to be multi-factorial and may reflect an increase in self-rated concerns regarding clinical performance or confidence as completion of training approaches. This conclusion is supported by the finding that across all surgical specialties 73.1 % of respondents believed a fellowship was necessary to obtain clinical competence for independent surgical practice. This implies either perceived shortcomings in the current surgical training curriculum or an over-estimation of the competence level required at the completion of formal training. It also implies a belief among respondents that fellowship training is able to impart the skills and knowledge required for independent surgical practice.

The relative proportions of those planning on undertaking a fellowship and those believing one to be necessary for attaining clinical competence is interesting to note and varies between specialties. Explanations for this disparity may relate to both the need to differentiate prior to consultant job applications and the relative difficulty in obtaining a consultant post. A period of additional training may be desirable in competitive jobs markets like neurosurgery and trauma and orthopedics, which have the highest overall levels of trainees intending to undertake a fellowship. Pediatric surgery on the other hand, a small specialty where training takes place in closely supervised regional centers, has the lowest overall levels of respondents intending to undertake a fellowship, potentially because of more closely aligned training needs and workforce requirements. The variation demonstrated illustrates that future approaches to quality assurance and administration of fellowships will need to reflect the needs of individual specialties rather than across surgery as a whole.

Independent of specialty, research fellows report the highest proportions of those both intending to complete a fellowship and believing fellowships are necessary to attain a level of clinical competence. Ellis et al. [7] have reported a correlation between those undertaking a period of dedicated research and future pursuit of fellowship training. This suggests that those undertaking research are more likely to undertake sub-specialist practice in future, or that those interrupting clinical training to undertake research recognize a need for additional clinical training time to ensure their own competence.

The similar fellowship intentions by gender contrast with recent US studies, which indicated that male residents, single residents, and residents without children were more likely to plan for fellowship training [8, 9]. It is interesting to consider whether this may reflect differences between the sexes in self-analysis of quality of training or perhaps their actual training, or whether lifestyle factors are the primary influence. It also raises the possibility that female surgical trainees may be disadvantaged by increasing levels of sub-specialization necessitating, or leading to, the formalization of fellowship training in surgery.

There was little support for making fellowships compulsory, with the majority of respondents favoring the existing processes. Trainees may enjoy the relative freedom of choice in fellowship training and fear that creating compulsory fellowships may limit their options and training opportunities. Current initiatives are underway through the surgical Royal Colleges to formalize fellowship schemes nationally to ensure quality assurance [1]. Overall, there was little support for formalization of the application process through national selection, with only a quarter of trainees in favor. This is despite the well-established and successful Board-administered fellowship matching schemes in place in the United States [10].

Variation in fellowship demand seen between specialties was positively correlated with the belief that fellowships are necessary to allow attainment of a level of clinical competence necessary for independent practice. This supports the primary self-reported reasons for undertaking a fellowship as increasing confidence, increasing competence, and allowing attainment of sub-specialist skills. Similar findings have been reported in North America, with residents worried about competence and skills more likely to plan for seeking fellowship posts [11].

Numerous studies have attempted to quantify the perceived benefit of fellowship training through changes in learning curves, and clinical and patient outcomes. These studies have almost exclusively reported favorable or at least neutral outcomes, raising the prospect of potential publication bias. In addition, many conclusions are limited by the retrospective study design, small numbers of fellowship-trained surgeons (often single-surgeon series), variable use of surrogate markers such as operative time alone, and a lack of appropriate comparison groups [12–14].

However, large retrospective studies with control groups have provided support for the fellowship model. Barbas et al. reported that colorectal or surgical oncology fellowship trained surgeons achieved significantly higher lymph node retrieval in subsequent colon cancer resections. It was acknowledged that superior technical expertise could contribute to this achievement, and that fellowship training could potentially act as a surrogate marker for subsequent oncological case volume [15]. Bianco et al. reported the largest retrospective analysis in this area, including 7,765 patients treated with radical prostatectomy by one of 72 surgeons over a 16-year period. Multivariate models were used to determine the learning curves for margin status and biochemical recurrence. Biochemical recurrence rates for fellowship-trained surgeons versus non-fellowship-trained surgeons did not differ for the first patients treated. Initial positive margin rates were superior for the fellowship-trained surgeons, although subsequent rates of positive margins improved similarly in both groups. The study concluded either that fellowship training conferred the ability to improve surgical technique or that surgeons who choose or who are appointed to a fellowship are those with a greater propensity to reflect on and improve their surgical technique. This raises the possibility that those undertaking fellowships may ultimately be a self-selected group, with fellowship a surrogate marker of favorable character traits associated with improved surgical performance [16].

Future research in this area should seek to assess the “value added” benefit of a fellowship post as an educational intervention in the surgical training pathway. The motivations to pursue a fellowship must also match the aims and curriculum of a post, given the reported differences between ideal and actual experiences [17].

The present study did not explore some of the negative aspects of fellowship training that have previously been highlighted, in particular the potential for a deleterious effect on the training of more junior colleagues. Few published studies tackle this controversial area, and all relate to experience in North America. Reported outcomes are mixed, with some studies suggesting co-existing fellowships have minimal impact on resident operative caseload [18, 19]. One study has associated the incorporation of fellowships with decreased procedural exposure by residents [20], and another demonstrated a significant increase in resident operative exposure following discontinuation of a fellowship [21]. A survey of urologists in one Canadian program demonstrated significant differences in opinion between residents and faculty regarding the impact of fellowship posts on resident training, with the residents believing that fellows “steal” operative cases [22]. These findings emphasize the need for quality assurance of fellowship posts to include a formal assessment of their effects on other trainees within the unit or wider training program.

Our results only offer a cross-sectional snapshot of current intentions regarding fellowship training, and these have previously been shown to vary with progression through the training process [23]. While the proportions of trainees planning to pursue fellowship training are similar to those reported in North America, the absence of previous UK studies in this area means that establishing chronological patterns is not yet possible. Longitudinal studies of resident training over the past two-decades in the United States report increases of 10–16 % in those opting to undertake fellowship training [5–7]. This sub-specialization may in the long-term have wider effects on the workforce, with greater specialization leading to a narrower breadth of individual service provision by surgeons. In the medium-term, this trend toward undertaking fellowship positions increases the complexity of work-force planning by prolonging the time spent in training prior to applying for substantive consultant positions.

## Conclusions

This study represents the first national pan-speciality survey to investigate the role of clinical fellowships among surgical trainees in the UK. Overall, three-quarters of current trainees intend to undertake a fellowship post. Variation in demand was positively correlated with the belief that fellowships are necessary to allow attainment of a level of clinical competence necessary for independent practice. There was little support among trainees for making fellowship training compulsory or for introducing national selection. The findings question whether the existing format of clinical training in surgery is able to deliver the necessary confidence and competence trainees require. The high proportions of trainees seeking periods of further sub-specialty training will also affect future workforce projections, both in terms of the breadth of services individual surgeons are able to provide and the increase in overall training time prior to entering consultant service. It is hoped that this analysis will aid future planning in this area and stimulate further studies into the perceived competence and confidence of surgeons completing their training programs.
